# Micro-RNA Expression Patterns Predict Metastatic Spread in Solid Pseudopapillary Neoplasms of the Pancreas

**DOI:** 10.3389/fonc.2020.00328

**Published:** 2020-03-13

**Authors:** Shmuel Jaffe Cohen, Michail Papoulas, Nadine Graubardt, Esther Ovdat, Shelly Loewenstein, Juliane Kania-Almog, Metsada Pasmanik-Chor, Eli Brazowski, Emanuela Cagnano, Ido Nachmany, Guy Lahat, Joseph M. Klausner, Nir Lubezky

**Affiliations:** ^1^Surgical Division Research Laboratory, Tel-Aviv Sourasky Medical Center Affiliated to the Sackler Faculty of Medicine, Tel-Aviv University, Tel-Aviv, Israel; ^2^Department of Surgery, Tel-Aviv Sourasky Medical Center Affiliated to the Sackler Faculty of Medicine, Tel-Aviv University, Tel-Aviv, Israel; ^3^Bioinformatics Unit, Tel-Aviv University, Tel Aviv, Israel; ^4^Tel-Aviv Sourasky Medical Center Affiliated to the Sackler Faculty of Medicine, Institute of Pathology, Tel-Aviv University, Tel-Aviv, Israel

**Keywords:** solid pseudopapillary neoplasm (SPN), metastasis, pancreatic cancer, microRNA, prognostic factors

## Abstract

Solid pseudopapillary neoplasm (SPN) of pancreas is a rare pancreatic neoplasm with a low metastatic potential. Up to 10% of patients with localized disease at presentation will develop systemic metastases, usually in the peritoneum or the liver. Due to the rarity of SPNs and the overall excellent prognosis, reliable prognostic factors to predict malignant biological behavior remain undetermined. Therefore, we aimed to define clinical, histological, and microRNA patterns that are associated with metastatic disease. We conducted a retrospective single center study on all patients operated for SPN of pancreas between 1995 and 2018. Clinical and pathological data were collected, and expression patterns of 2,578 human microRNAs were analyzed using microRNA array (Affimetrix 4.1) in normal pancreases (NPs), localized tumors (LTs), and metastatic tumors (MTs). The diagnosis of SPN was confirmed in 35 patients who included 28 females and 3 males, with a mean age of 33.8 ± 13.9 years. The only clinical factor associated with metastases was tumor size (mean tumor size 5.20 ± 3.78 in LT vs. 8.13± 1.03 in MT, *p* < 0.012). Microscopic features of malignancy were not associated with metastases, nor were immunohistochemical stains, including the proliferative index KI67. Higher expressions of miR-184, miR-10a, and miR-887, and lower expressions of miR-375, miR-217, and miR-200c were observed in metastatic tissues on microarray, and validated by real-time polymerase chain reaction. Hierarchal clustering demonstrated that the microRNA expression pattern of MTs was significantly different from that of LTs. The only clinical factor associated with metastases of SPN of pancreas was tumor size. Histological features and immunohistological staining were not predictive of metastases. A panel of six microRNAs was differentially expressed in MTs, and these findings could potentially be used to predict tumor behavior. Validation of these results is needed in larger series.

## Introduction

Solid pseudopapillary neoplasms (SPNs) of the pancreas were initially described by Frantz in 1959 (1). The frequency of their detection and diagnosis have been steadily increasing, with more than 60% of the total of known cases having been reported in the last 10 years (2, 3). Most SPNs are found in young female patients, and they are characterized by being localized and large in size, with solid and cystic components. Complete, margin negative surgical resection is considered curative in most cases (4–10). However, up to 15–20% of patients demonstrate gross malignant features, such as invasion of adjacent organs or distant metastases, at the time of diagnosis (3, 11–15). Moreover, up to 10% of patients with localized disease at presentation will develop systemic metastases, usually in the peritoneum or the liver (3). Large tumor size, male gender, high proliferative index assessed by Ki-67 immunoreactivity, and microscopic malignant features were reported to correlate with malignant behavior, however data on the sensitivity and specificity of these factors are limited (2). Due to the rarity of SPNs, and the overall excellent prognosis, reliable prognostic factors to predict malignant biological behavior remain undetermined.

MicroRNAs are small non-encoding RNAs that are cleaved from 70 to 100 nucleotide pre microRNA precursors in the cytoplasm into their mature form of 19 to 25 nucleotides (16, 17). Single-stranded microRNAs bind messenger RNAs of potentially hundreds of genes at the 3′ untranslated region with perfect or near-perfect complementarity, resulting in degradation or inhibition of the target messenger RNA. In humans, aberrant expression of microRNAs contributes to carcinogenesis by promoting the expression of proto-oncogenes or by inhibiting the expression of tumor suppressor genes. Such “oncomicroRNAs” have been demonstrated in a variety of hematologic and solid malignancies (18), but their role in the progression of SPN has not been studied.

We performed an in-depth clinical, histological, and microRNA (miRNA) analysis of a cohort of patients with localized and metastatic SPN. Our aim was to define clinical and histological risk factors for metastasis in SPN, and to identify miRNAs that are differentially expressed in metastatic tumors compared with localized tumors.

## Materials and Methods

### Tissue Samples

All patients undergoing pancreatectomy in a single tertiary university-affiliated medical center from 1995 to 2018 were retrospectively reviewed, and those with a pathological diagnosis of SPN of the pancreas were included in the current analysis. Diagnosis of SPN was confirmed in 37 cases. Electronic medical records were reviewed for the demographic, radiologic, and perioperative details. Histological slides were reviewed by a single pathologist (E.B.) to confirm the diagnosis of SPN, and to assess histological features of aggressive biological behavior, including cellular atypia, capsule invasion, lymph node metastasis, lymphovascular invasion, perineural invasion, and peripancreatic fat tissue invasion. Immunohistochemical studies (IHC) were performed in 37 specimens, and they included staining for beta-catenin, CD10, neuroendocrine markers (neuron-specific enolase, chromogranin A, and synaptophysin), progesterone, vimentin, Ki-67, and cytokeratin (AE1/AE3) (15).

Four patients with metastatic disease, either at presentation (*n* = 1) or during follow-up (*n* = 3) were identified. Metastatic patients were matched for gender and tumor size with six patients who had localized disease. Tissues for miRNA analysis were obtained from the surgical specimens, and they included tissues from localized tumors (LTs), metastatic tumors (MTs), and normal pancreatic tissues (NTs).

### miRNA Microarray

For the miRNA microarray experiments, formalin-fixed paraffin-embedded blocks of SPN specimens were obtained from the archives of the medical center's Institute of Pathology. The samples were reviewed, and ten micrometer-thick sections were cut from the tumor block and transferred onto glass slides. A single glass slide containing a block sample was stained with hematoxylin and eosin, and tumor margins were marked by the pathologist (E.B.). Microdissection was performed manually to extract tumoral tissue with a sharp scalpel. Total RNA, including miRNA, was extracted using an microRNeasy formalin-fixed paraffin-embedded kit (Qiagen, Hilden, Germany). Total RNA concentrations were measured using a NanoDrop ND-1000 spectrophotometer (NanoDrop, Wilmington, DE, USA) (19). The miRNA microarray experiments were utilized for microRNA hybridization using GeneChip microRNA 4.1 Array strip (Affymetrix) (a total of 11 arrays). Samples (500 ng) were labeled with the Genisphere FlashTag Biotin Labeling Assay, which utilizes the 3DNA technology. The 3DNA dendrimers are ligated to samples to allow multiple biotins (~15) to bind to each poly-A-tailed RNA molecule. After FlashTag ligation, the samples were hybridized overnight on the Affymetrix GeneChip microRNA array. The arrays were washed and immediately scanned with a GenePix 4000B array scanner (20).

### Microarray Analysis

Bioinformatics Analysis of miRNA profiles were extracted from Affymetrix CEL files using Partek Genomics Suite (Partek GS 6.5; http://www.partek.com; Partek Inc., St. Louis, MO, USA). Data were normalized and summarized with the Robust Multichip Average algorithm and converted to log2 values and the data were used for statistical analysis. One-way ANOVA was performed to test for significant differences between the means of the analyzed groups. miRNA expression data were sorted using cutoffs of *p* < 0.05 under false discovery rate (FDR) correction for multiple comparisons adjustment criteria and a fold difference of two. The panel of the most significantly differentially expressed miRNAs (i.e., a fold change of more than 2 or <-2 and *p*[FDR] < 0.05) were determined and selected for further validation (20). Hierarchical cluster analyses were performed using the Pearson correlation with Ward's method.

### Quantitative Reverse Transcription Real-Time PCR (RT-PCR)

Total RNA from formalin-fixed paraffin-embedded blocks of SPN specimens were extracted as described above. Total RNA concentrations were measured using a NanoDropOne spectrophotometer (ThermoFisher Scientific, Madison, WI, USA). Quantitative analyses of miRNA levels in SPN samples were performed using the stem-loop TaqMan MicroRNA Assays kit (Applied Biosystems, Foster City, CA, USA). The Taq-Man microRNA assays follow a 2-step protocol involving reverse transcription with human mature miRNA-specific primers, followed by real-time PCR with TaqMan probes. These assays target only the mature microRNA sequence, and the precursors are not detected. Briefly, using 10 ng of total RNA, mature miRNA was reverse-transcribed into cDNA with mature microRNA-specific looped RT primers from the TaqMan MicroRNA Assays kit and reagents from the TaqMan MicroRNA Reverse Transcription kit (Applied Biosystems) following the manufacturer's directions. RT-PCR was performed on the cDNA with Applied TaqMan™ Fast Advanced Master Mix for each miRNA of interest following the manufacturer's directions. Triplicate reactions were incubated in an Applied Biosystems StepOnePlus Real-Time PCR System in a 96-well plate for 20 s at 95°C, followed by 40 cycles for 1 s at 95°C and 20 s at 60°C. For each sample, the threshold cycle (Ct) was calculated by the ABI StepOne Sequence Detection System software v2.3. The non-coding small nuclear RNA U6 (U61973; Applied Biosystems) was used as internal control. Gene expression levels were quantified using the ABI StepOne Software v2.3, and relative fold expression was calculated using the comparative C_t_ (2^−ΔΔCt^) method for relative quantitation of gene expression. Statistical analysis was performed to provide standard deviations for comparisons of gene expression between samples, as well as *p*-values from *t*-tests for comparison between biological groups (19).

### miRNA Target Network and Pathway Enrichment

miRNAs were analyzed by the miRNet tool for gene targets, miR-gene network and pathway and function enrichment (https://www.mirnet.ca/) in order to investigate the potential target of miR-184, miR-10a, miR-887, miR-375, miR-217, and miR-200c.

### Ethical Considerations

The study protocol was approved by the Human Ethics Review Committee of the Israel Ministry of Health and the Tel-Aviv Medical Center (19).

### Statistical Analysis

Excel or GraphPad Prism Software Version 5 was used for statistical analysis. The statistical significance of the differences between two groups was estimated by an unpaired two-tailed (one-tailed when stressed) *T*-test. A *p* < 0.05 was taken as significant. Outliers that were above (mean + 2^*^SD) and below (mean −2^*^SD) were excluded.

## Results

### General Characteristics of Resected SPNs

The diagnosis of SPN was confirmed in 35 patients, most of the patients were females (86%), the mean age of the cohort was 33.8 ± 13.7 years (range 18–64 years). The most common location of the tumor was the body or tail of the pancreas, and the mean tumor diameter was 5.20 ± 3.78 cm (range 0.9–14 cm). The surgical procedures included distal pancreatectomy (*n* = 25, 71.4%), and radical pancreaticoduodenectomy (*n* = 5, 14.3%), and tumor enucleation (*n* = 2, 5.7%). Gross invasive features were present in two patients, and they included invasion to the superior mesenteric artery and invasion to the left colon. Pathological confirmation of SPN was according to the WHO histologic criteria (21). Immunohistological studies were performed in the 35 study patients to confirm the diagnosis of SPN and to further characterize the tumor. All of the tumors showed negative membranes and positive nuclear staining of beta-catenin.

Four patients had metastatic SPN. One patient was diagnosed with simultaneous pancreatic tumor and liver metastases. Three additional patients were initially diagnosed with localized SPN and subsequently developed a liver (*n* = 2) or a peritoneal and lymphatic (*n* = 1) recurrence during follow-up. Time from resection of the primary tumor to diagnosis of recurrence was between 1 and 7 years.

### Clinical Risk Factors for Metastatic Disease

**Figure 1:**
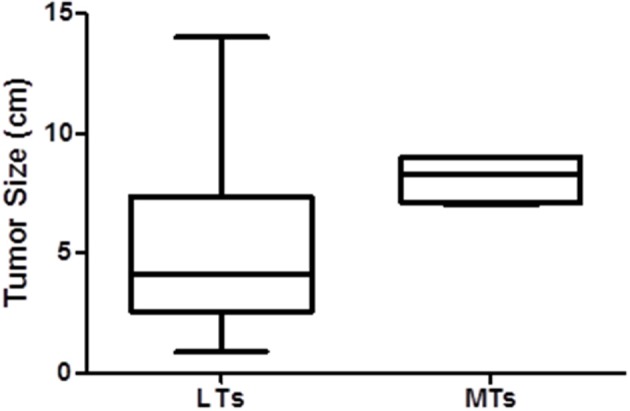
The difference in tumor size between MT and LT SPNs displayed by a boxplot.

The clinical features of patients with metastatic and localized disease are provided in Table 1. All of the patients with MTs were females with a mean age of 35.5 ± 19.3, similar to the patients with LTs. The patients with MTs did not have any distinguishing symptoms at presentation. All of the MTs were located in the pancreatic body or tail. The only significant difference between the groups was that tumor size was significantly larger in the patients with MTs compared to those with LTs (mean 5.20 ± 3.78 cm, range 0.9–14 cm vs. 8.13 ± 1.03 cm, range 7–9 cm, *p* < 0.012) also shown by boxplot (Figure 1).

**Table 1:** Clinical features of patients, pathological and Immunohistological profile of metastatic and localized SPNs.

	**Localized SPN** **(*N* = 31)**	**Metastatic SPN** **(*N* = 4)**
**Clinical Features**		
**Gender**		
Male	3	0
Female	28	4
Age, y	33.8	35.5
**Symptoms at Presentation**		
Abdominal pain	13	2
Back pain	3	0
Weight loss	6	0
Incidental	11	0
Fever of unknown origin	2	0
**Tumor Location**		
Head	7	0
Tail	15	3
Body/neck	9	1
Tumor size (range)	5.20 cm (0.9–14)	8.12 cm (7–9)
**Type of Surgery**		
Enucleation	2	0
Distal pancreatectomy	22	3
Pancreaticoduodenectomy	4	1
Adjacent organ invasion	2	0
**Pathological and Immunohistological Profile**		
Resection margin	Free = 31	Free = 3, unknown = 1
**Microscopic Malignant Features**		
Cellular atypia	6	2
Capsule invasion	8	1
Peripancreatic fat invasion	4	1
Perineural invasion	2	1
Lymphovascular invasion	3	1
Lymph node metastases	0	0
**Immunohistological Staining (Positive/Tested)**		
Beta catenin	17/17	2/2
CD-10	20/22	1/1
Neuron-specific enolase	14/14	1/1
Chromogranin A	4/28	0/3
Synaptophysin	17/25	3/3
Progesterone	10/17	1/3
Vimentin	18/18	2/2
KI-67 ≥ 3%	2/23 (5%, 10%)	1/3 (3%)
Cytokeratin	10/12	3/3

### Histological Risk Factors for Metastatic Disease

The pathological and immunohistological characteristics of localized and metastatic SPNs are listed in Table 1. The histological features of malignancy, including cellular atypia, capsule invasion, peripancreatic fat invasion, perineural invasion, and lymphovascular invasion, were not predictive of metastatic spread. Immunohistochemical staining was used to confirm the diagnosis of SPN, and no immunohistological stains were predictive of malignant behavior. The proliferative index KI67 was performed in three metastatic and 23 localized tumors, and it also was not predictive of malignant behavior.

### miRNA Expression Patterns of Metastatic Disease

**Figure 2:**
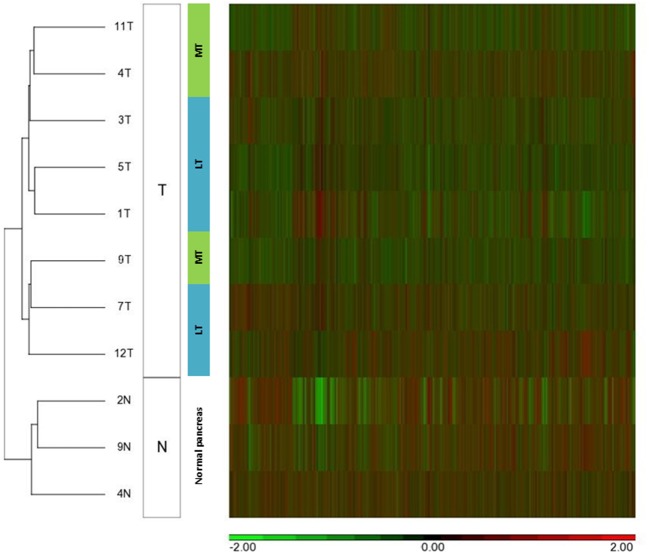
MicroRNA expression patterns of metastatic SPN disease. Unsupervised hierarchical clustering analysis of microRNA expression of 5 localized tumors (LT), 3 metastatic tumors (MT), and 3 normal pancreatic tissues (N). SPN tumors (T) were clustered based on microRNA expression patterns (columns).

**Figure 3:**
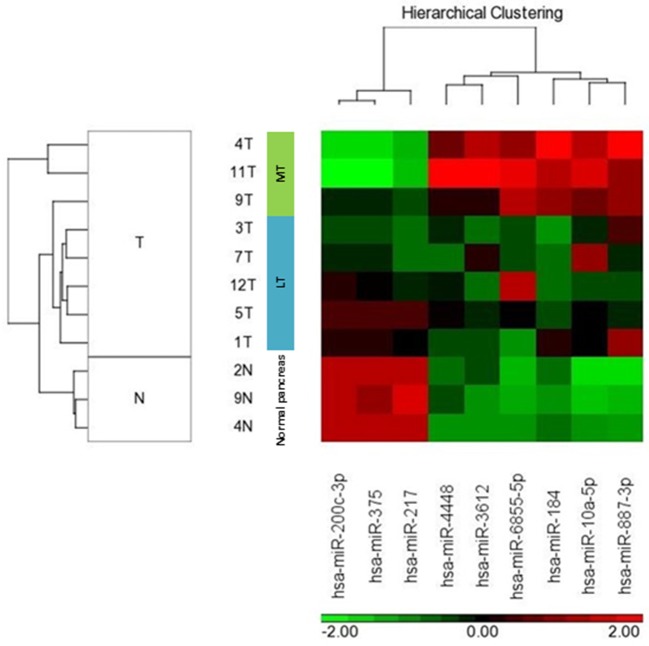
MicroRNA expression of localized and metastatic SPN classified samples. HeatMap and unsupervised clustering schema of 5 localized tumors (LT), 3 metastatic tumors (MT), and 3 normal pancreatic tissues. The normalized expression level (h) of each microRNA is color coded.

We compared miRNA expression in available metastatic SPN tissue (*n* = 3) and localized SPN (*n* = 5). We assessed the distribution of microRNA expression for each of the 2,578 mature miRNAs using the Affymetrix microRNA 4.1 array strip, and the correlations between the expression levels of the microRNAs (Figure 2). Nine miRNA expressions were significantly differentially expressed between metastatic (*n* = 3) and localized (*n* = 5) groups, with a fold change of two and more and a *p* < 0.05 (Table 2). The significant differences in microRNA expression were derived from differences in the metastatic group (*n* = 3) compared to the normal tissue group (*n* = 3) and to the LT tissue group (*n* = 5), as shown in a heat map and a clustering schema (Figure 3). The expression of *miR-217, miR-200c*, and *miR-375* was significantly lower, whereas the expression of *miR-10a, miR-887, miR-184, miR-6855, miR-3612, and miR-4448* was significantly higher in the MTs compared with the LTs (Table 2). Hierarchical clustering by means of the expression levels of these microRNAs demonstrated that they distributed into three distinct groups.

**Table 2:** MicroRNAs with different expression patterns in localized and metastatic SPN.

**Transcript ID** **(array design)**	***p*-value**	**Fold change**	**Fold change** **(description)**
hsa-miR-217	0.035	−4.751	MT down
hsa-miR-10a-5p	0.014	3.818	MT up
hsa-miR-887-3p	0.016	2.229	MT up
hsa-miR-184	0.001	7.342	MT up
hsa-miR-200c-3p	0.012	−11.673	MT down
hsa-miR-375	0.017	−14.178	MT down
hsa-miR-6855-5p	0.022	2.652	MT up
hsa-miR-3612	0.009	2.350	MT up
hsa-miR-4448	0.035	2.554	MT up

*Significantly differentially expression of 9 microRNAs with a fold change of 2 and more MTs (N = 3) and LTs (N = 5)*.

*MT, metastatic tumor; LT, localized tumor*.

### miRNA Signature as Prognostic Markers of Clinical Outcome

The prognostic value of six miRNAs was assessed by real-time qPCR. miRNA sequences were selected according three parameters: signal intensity, fold-change, and *p*-value. The same samples for validation experiments were used as in the original microarray experiment. Test samples were collected from exactly the same RNA extraction preparation as that used for the original microarray experiment. We analyzed five LT cases, three MT cases, and three NT samples.

**Figure 4:**
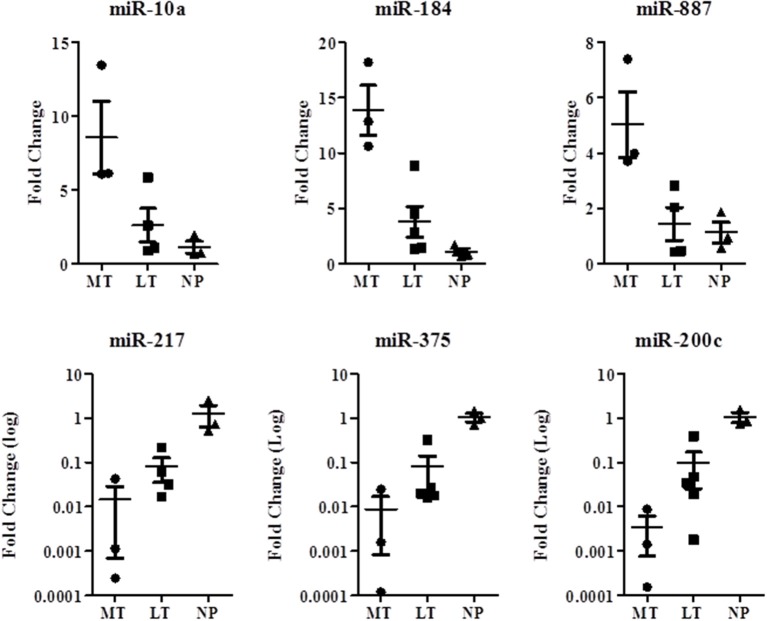
MicroRNAs microarray validation study for comparison between localized and metastatic SPN microRNAs. Quantitative reverse transcription real-time PCR was performed to formalin-fixed paraffin-embedded blocks of SPN RNA extracted specimens from 5 localized tumors (LT) (n), 3 metastatic tumors (MT), and 3 normal pancreatic tissues (NT). *P* < 0.05.

The microRNA expression level of *miR-217, miR-200c*, and *miR-375* was higher in the LTs and NPs compared with the MTs. The microRNA expression level of *miR-10a, miR-887*, and *miR-184* was higher in the MTs compared with the LTs and NPs (Figure 4). MicroRNA expression was significantly associated with MTs, and showed the same trend as that observed in the micro-array results. miRNA-184, miR-217, miR-375, and miR-200c were differentially expressed in LT compared with NP (Table 3).

**Table 3:** Validation of differentially expression of six microRNAs in metastatic (*n* = 3) and localized tumors (*N* = 5).

	***p*-value**	**Significance**	***N* = MT**	***N* = LT**	**Fold change** **MT vs. NP**	**Fold change** **LT vs. NP**	**Description**
miR-10a	0.027	*	3	4	8.552	2.608	UP
miR-184	0.010	*	3	5	13.879	3.801	
miR-887	0.027	*	3	4	7.323	3.599	
miR-217	0.041	*	3	4	0.015	0.081	Down
miR-375	0.030	*	3	5	0.009	0.080	
miR-200c	0.035	*	3	5	0.003	0.099	

*Unpaired one-tailed T-test, p < 0.05. Outlier that were above (Mean + 2*SD) and any points below (Mean −2*SD) were excluded.

*MT, metastatic tumor; LT, localized tumor; NP, normal pancreas*.

### Six Human MicroRNA Targets and Network and Pathway Enrichment

**Figure 5:**
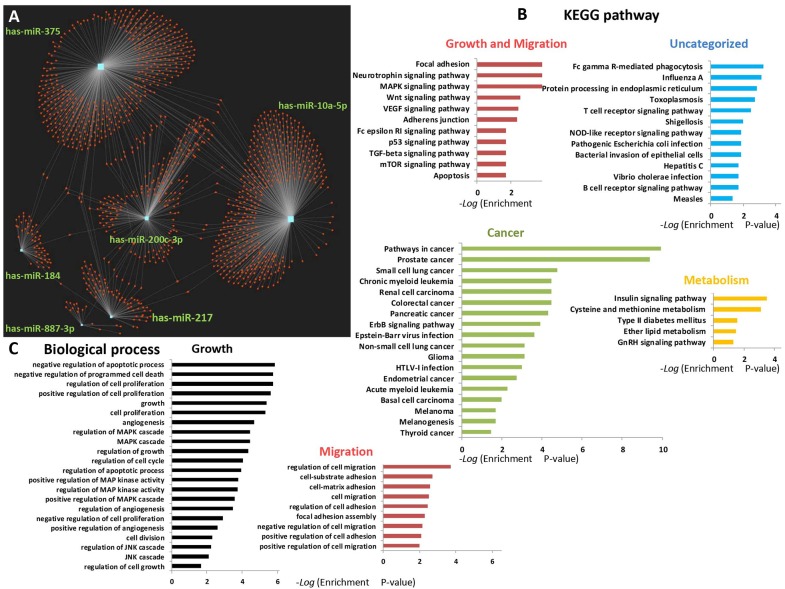
Network and pathway enrichment of 6 human miRNA targets. Six selected miRNAs (miR-10a, miR-887, miR-184, miR217, miR375, and miR-200C) were analyzed by miRNet tool for gene targets, and miR-gene network, pathway, and function enrichment (https://www.mirnet.ca/). **(A)** 1,306 gene targets for 6 miRNAs are presented. **(B)** Enriched pathways for migration, cancer, metabolism, and uncategorized. **(C)** Enriched biological processes for migration and growth. The KEGG analysis was performed on all 47 significantly enriched pathways. The biological process analysis includes functions of interest extracted from 242 significant results (*p* < 0.05).

A bioinformatics analysis that was used to elucidate the predicted biological processes and pathways of miR-184, miR-10a, miR-887 (up-regulated), and miR-375, miR-217, miR-200c (down-regulated) targets and network are presented in Figure 5. An miRNet tool (https://www.mirnet.ca/) for microRNA-gene target prediction was used to predict the targets and to draw the network. A total of 1,306 gene targets were found (Figure 5 and Supplementary Excel Datasheet File). Specifically, miR-10a targeted 463 different genes (35.5%), miR217 targeted 80 genes (6.1%), miR-184 targeted 55 genes (4.2%), miR-200c targeted 214 genes (16.4%), miR-375 targeted 477 genes (36.5%), miR-887 targeted 17 genes (1.3%). Kyoto Encyclopedia of Genes and Genomes (KEGG) pathway analysis enrichment was performed for the six microRNAs targets and revealed 47 significantly enriched pathways. Enriched pathways for growth and migration (red), cancer (green), metabolism (yellow), and uncategorized (blue) are displayed in Figure 5B. Twenty-nine of the pathways (62%) were connected directly to cancer and 11 (23%) were associated with migration, proliferation, and cell growth. The 10 most significantly enriched pathways were those of migration, growth, and cancer.

Focal adhesion, neurotrophin signaling, MAPK signaling, and ErbB signaling were the most significantly enriched specific pathways (Figure S1A and an Supplementary Excel Datasheet File display all 47 of the enriched pathways).

An enrichment analysis of the biological processes of the six miRNAs targets was performed and exhibited functions related to migration and growth (Figure 5C extracted from 242 significant results, Figure S1B, and in an Supplementary Excel Datasheet File). Cell growth proliferation and migration were significantly enriched in 31 biological processes (13%).

## Discussion

SPNs are generally considered benign tumors. However, it is well-recognized that up 20% of these tumors behave aggressively and exhibit local invasion or systemic metastases (3, 11–14). Defining prognostic factors that can predict tumor recurrence after complete resection is important for patient counseling, planning long-term surveillance, and potentially developing therapeutic targets. Our cohort of 35 patients with SPN included two patients with local invasive tumors and four patients who developed systemic metastases. Since both of the patients that had local invasive tumors did not develop either local or systemic recurrence after margin-negative resection, we focused upon defining clinical, pathologic and molecular risk factors that are associated with systemic metastases, and not local tumor invasion.

The only clinical factor associated with metastatic disease was tumor size (8.13 cm in MTs and 5.20 cm in LTs, *p* < 0.012). Tumor location, presenting symptoms, gender, age and preoperative endoscopic ultrasound-guided biopsy were not associated with metastatic disease. The largest reported cohort of SPN by Kang et al. (3) included 351 patients with SPN from 17 medical institutions in Korea. Only nine patients (2.6%) sustained tumor recurrence, which is significantly lower than our recurrence rate of 10%. Those authors reported that the only clinical factor associated with tumor recurrence on multivariate analysis was a tumor size large than 8 cm, in support of our findings.

We performed a comprehensive review of tumor histology to assess microscopic features associated with invasiveness, including cellular atypia, capsule invasion, peripancreatic fat invasion, lymph node metastases, perineural invasion, and lymphovascular invasion (15). None of these histological features was associated with risk of systemic metastases. Our results are in contrast with those reported by Kang et al. (3). They found that individual pathologic components of malignancy were not clinically significant in predicting recurrence of resected SPNs, however, having any microscopic features of malignancy was associated with risk of recurrence on multivariate analysis.

In an attempt to define molecular markers that are associated with metastatic disease, we performed a high-throughput microRNA array of tissues extracted from MTs, LTs, and NTs. The results showed that *miR-10a, miR-887*, and *miR-184* were expressed at a higher level in MTs, and that *miR-375, miR-200c, and miR-217* were expressed at a lower level in MTs. Hierarchical clustering by means of these microRNAs yielded their division into three distinct groups. Such microRNA signatures could be used to identify tumors that are at increased risk to develop metastases, thereby requiring long-term surveillance. Moreover, these microRNAs may also have a role in tumor progression, and could serve as potential therapeutic targets.

We reviewed the literature on the microRNAs that were found to be differentially expressed in MTs. *miR-184* was abnormally expressed in various tumor cells, including pancreatic ductal adenocarcinoma (22). Down-regulation *miR-184* was shown to inhibit cell proliferation, lower invasion, and cause up-regulation of apoptotic protein *caspase-3* in transfected PANC-1 cells (22). SPNs are characterized by increased levels of phosphorylated β*-catenin*. Du et al. (23) showed that the inhibition of miR-184 may reduce the tumor volume of osteosarcomas via regulation of the *Wnt/*β*-catenin* signaling pathway. Over-expression of miR-184 leads to increased levels of phosphorylated β*-catenin* (23), suggesting that the role of miR-184 in promoting systemic metastases in SPN is associated with activation of the *Wnt/*β*-catenin* signaling pathway.

The reported effects of *miR-10a* overexpression in cancer metastasis were contradictory (24). Overexpression of *miR-10a* was involved in the invasive potential of pancreatic cancer cells partially via suppression of HOXA1(25). In non-pancreatic cancers, *miR-10a* enhanced the metastatic potential of cervical cancer cells (26), and was markedly upregulated in primary tumor tissues in patients with positive lymph node metastasis (27). *miR-10a* enhanced the migration and invasion of SW480 and SW620 cells in colorectal cancer metastasis (24) but, in contrast, suppressed the formation of liver metastases in nude mice. Also in contrast to the reported effect of miR-184 on the *Wnt/*β*-catenin* signaling pathway (26) are the findings that overexpression of miR-10a reduced β-catenin at both protein and transcription levels, while pretreatment with the Wnt signaling activator Licl partially attenuated the suppression effects of miR-10a overexpression on osteoblast differentiation and angiogenesis (24).

miR-887 was demonstrated to have a role in pancreatic cancer. The expression of miR-887 in pooled tissue extracts of 10 pancreatic ductal adenocarcinoma (PDAC) patients was downregulated (28) compared to normal pancreatic tissue. miR-375 was also downregulated in a variety of cancers, and low expression of miR-375 was shown to be a negative predictive factor in several cancers, including pancreatic cancer (29). There are a number of reports on the role of miR-217 down-regulation in promoting aggressive tumor behavior. *miR-217* down-regulation in pancreatic cancer was associated with the elevation of *E2F3* gene a transcription factor family, which plays an important role in cellular proliferation, apoptosis, and differentiation (30). In a recent transcriptome-wide association study, the expression level of *E2F3* was identified as one of 19 genes with significant pancreatic tumor association and pancreatic cancer progression (31).

To the best of our knowledge, there are no data in the “The Cancer Genome Atlas” (TCGA) database to confirm our study findings. KEGG analysis for the six miRNAs revealed 47 significantly enriched pathways, of which the four top-scoring modified pathways that affect migration and cell growth were: focal adhesion, neurotrophin signaling, MAPK signaling, and ErbB signaling (32–35). Those miRNA target functions were also significant in the GO biological process (BP) enrichment analysis, with 31 BPs (13% of all significant BPs) involved in cell migration, cell proliferation, and growth processes.

The impact of our detected miRNAs on cell migration and growth was studied in pancreatic, prostate, and cervical cancer cell lines and shown to affect cell migration and growth. For example, miR-10a (36), miR-184 (22), miR-200c (37) affected cell migration and growth in pancreatic cells, miR-217 affected cell growth in a colorectal cancer cell line (30), miR-887 affected cell growth in a prostate cancer cell line (38), and overexpression of miR-375 suppressed cervical cancer cell proliferation, migration, invasion, and angiogenesis, and the inhibition of miR-375 expression significantly enhanced these functions *in vitro* (39). The research of SPNs cells *in vitro* is challenging due to the unclear origin of their neoplasm, despite numerous investigations (40). It is, therefore, not feasible to confirm the biological function of differentially expressed miRNAs in SPN tumor cells by means of *in vitro* studies. Additional studies are necessary to first elucidate the origin of the neoplasm and then to design research tools for the investigation of SPN cells *in vitro*.

Li et al. (41) identified potential biomarkers which differentially diagnose solid pseudopapillary tumors and pancreatic malignancies. Down-regulation of miR-200c was one of 14 genes that discriminated SPNs from pancreatic malignancies, including pancreatic neuroendocrine tumors and pancreatic ductal adenocarcinoma.

The expression levels of each miRNA were normalized to the expression level in normal pancreatic tissue in our validation studies. The change in expression levels was expressed as fold change in the LT or MT compared with normal pancreatic tissue. We also compared miRNA expression levels in LTs compared with MTs, and the results revealed a pattern of progression in miRNA expression levels in NP, LT, and MT in all six miRNAs, as depicted in Figure 4.

The main limitations of this study are the retrospective retrieval of the data and the limited number of patients. Nevertheless, SPNs are rare tumors, and MTs are very rarely diagnosed. Only four metastatic SPN patients were treated at our center. Notably, only 10 patients with metastatic SPN were included in the largest reported series of 352 resected SPNs, (3). Further validation of these data in a larger cohort of patients with MTs and LTs is warranted.

In conclusion, our study confirms that 10% of SPN patients demonstrate malignant features and develop metastatic disease. The only clinical factor associated with metastases was tumor size. Histological features and immunohistological staining were not predictive of metastases. A panel of six microRNAs, including *miR-184, miR-10a, miR-887, miR-217, miR-200c, and miR-375* were differentially expressed in MTs, and could potentially be used to predict tumor behavior. These findings may be contributory to establishing more focused guidelines for patient management and to the search for more effective treatment options.

## Data Availability Statement

The datasets generated and analyzed during the current study are available in the NCBI Gene Expression Omnibus GEO database (accession number: GSE140719).

## Ethics Statement

The studies involving human participants were reviewed and approved by Human Ethics Review Committee of the Israel Ministry of Health and the Tel-Aviv Medical Center. The ethics committee waived the requirement of written informed consent for participation.

## Author Contributions

NL conceived and designed the study and acquired and interpreted data. NL and SC drafted the manuscript. SC analyzed the data, wrote, designed results, figures, and tables. JK-A and MP performed the validation study. EO designed Table 1. MP, NG, and SL performed the microarray study. MP-C performed the microarray analysis and bioinformatics analysis of biological processes and pathways. EB and EC performed the clinical and pathological aspects. IN and GL reviewed the manuscript. JK critically revised the manuscript.

### Conflict of Interest

The authors declare that the research was conducted in the absence of any commercial or financial relationships that could be construed as a potential conflict of interest.

## Supplementary Material

Figure S1 Network and pathway enrichment of six human miRNA targets. Six selected miRNAs (miR-10a, miR-887, miR-184, miR217, miR375, and miR-200C) were analyzed by miRNet tool for gene targets, as well as miR-gene network, pathway, and function enrichment (https://www.mirnet.ca/). **(A)** KEGG analysis. **(B)** Biological process analysis (*p* < 0.05).

Supplementary Excel Datasheet File Network and pathway enrichment of six human miRNA targets.
