# Supplementary figures and images for: Micro-RNA Expression Patterns Predict Metastatic Spread in Solid Pseudopapillary Neoplasms of the Pancreas
Source: Front Oncol. 2020 Mar 13;10:328. doi: 10.3389/fonc.2020.00328 (PMC7082878; doi:10.3389/fonc.2020.00328)

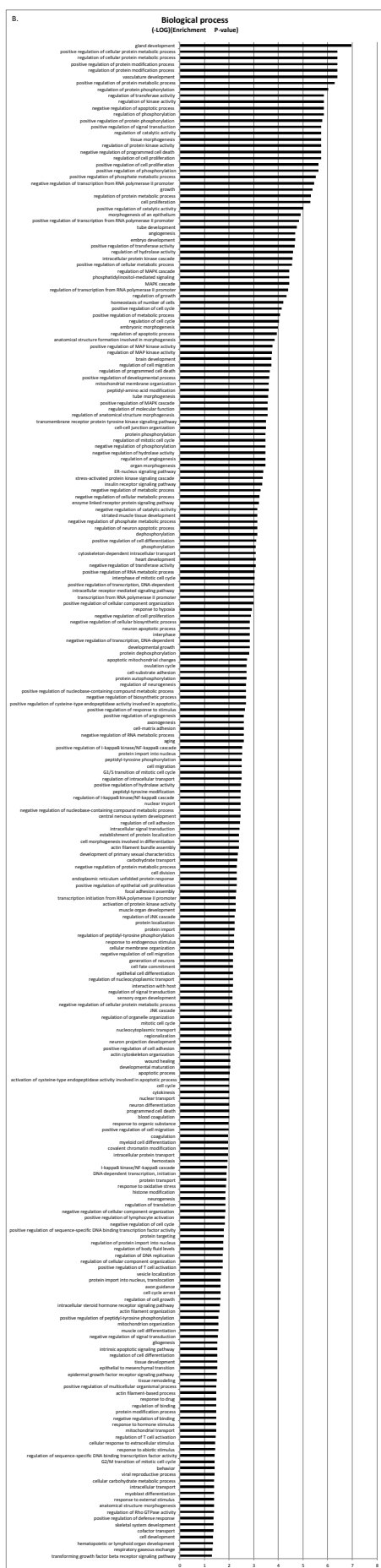

Supplement: Supplementary file 1 [file Data_Sheet_2.PDF]

A.

# KEGG Pathway (-LOG)(Enrichment P-value)

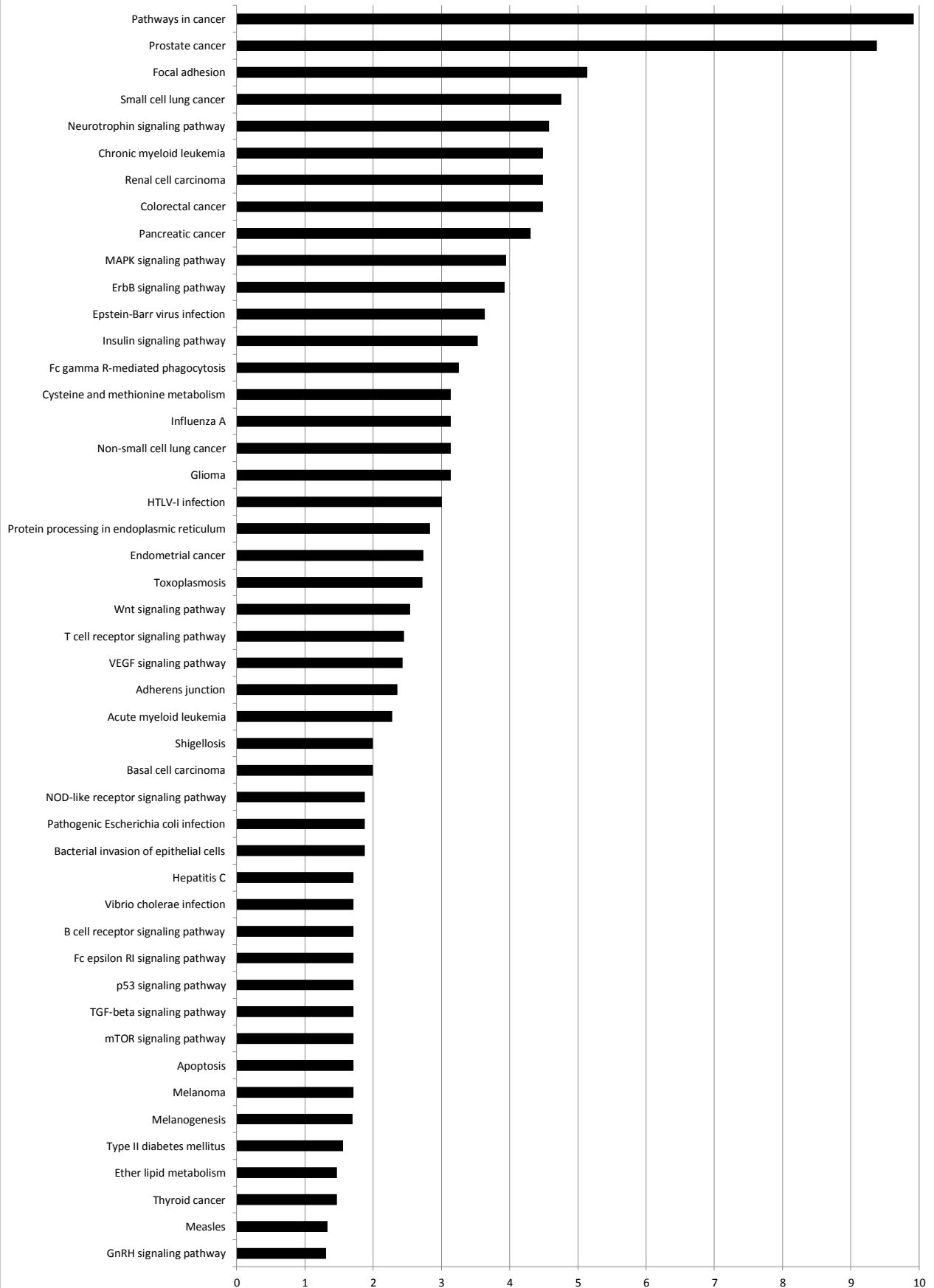

Supplement: Figure S1 — Network and pathway enrichment of six human miRNA targets. Six selected miRNAs (miR-10a, miR-887, miR-184, miR217, miR375, and miR-200C) were analyzed by miRNet tool for gene targets, as well as miR-gene network, pathway, and function enrichment (https://www.mirnet.ca/). (A) KEGG analysis. (B) Biological process analysis (p < 0.05). [file Data_Sheet_1.PDF]
